# Supplementary material for: Targeting BRF2 in Cancer Using Repurposed Drugs
Source: Cancers (Basel). 2021 Jul 27;13(15):3778. doi: 10.3390/cancers13153778 (PMC8345145; doi:10.3390/cancers13153778)
Supplement: Supplementary file 1 [file cancers-13-03778-s001.zip › Analysis/BRF2_1b_Chemosensitivity_proliferation_stemness_autophagy_pancan_heatmap3_allstats.pdf]

Chemosensitivity, proliferation, stemness and autophagy (BRF2)

Cancer type

	ACC	BLCA	BRCA	CESC	CHOL	COAD	DLBC	ESCA
Autophagy−related prognostic signature	r=−0.0579 p=0.6121 n=79	r=0.0189 p=0.7038 n=408	r=−0.1477 p=8.3e−07 n=1104	r=−0.0708 p=0.2162 n=307	r=−0.2329 p=0.1716 n=36	r=−0.0794 p=0.0913 n=453	r=−0.2160 p=0.1404 n=48	r=−0.0078 p=0.9166 n=185
Stemness (mRNA)	r=0.0457 p=0.6947 n=76	r=0.0081 p=0.8721 n=400	r=0.1303 p=1.9e−05 n=1072	r=0.0761 p=0.1954 n=291	r=0.2257 p=0.1857 n=36	r=0.0191 p=0.6919 n=434	r=NA p=NA n=NA	r=0.2738 p=0.0002 n=182
Stemness (DNA methylation)	r=0.0471 p=0.6859 n=76	r=0.0399 p=0.4261 n=400	r=0.0167 p=0.6430 n=771	r=0.2001 p=0.0006 n=291	r=−0.2631 p=0.1210 n=36	r=0.0522 p=0.3771 n=288	r=NA p=NA n=NA	r=0.2557 p=0.0005 n=182
Predicted pCR to T/FAC chemotherapy	r=0.0030 p=0.9794 n=77	r=−0.0601 p=0.2281 n=404	r=−0.1048 p=0.0005 n=1085	r=0.0070 p=0.9051 n=294	r=−0.0786 p=0.6486 n=36	r=0.0078 p=0.8693 n=444	r=−0.3891 p=0.0063 n=48	r=0.0257 p=0.7288 n=184
Proliferation score	r=0.1893 p=0.1014 n=76	r=−0.0027 p=0.9567 n=393	r=0.0856 p=0.0051 n=1072	r=0.0462 p=0.4329 n=290	r=−0.1244 p=0.4764 n=35	r=−0.0048 p=0.9213 n=434	r=−0.1281 p=0.3854 n=48	r=0.1840 p=0.0154 n=173

	GBM	HNSC	KICH	KIRC	KIRP	LGG	LIHC	LUAD
Autophagy−related prognostic signature	r=−0.0911 p=0.2578 n=156	r=0.0896 p=0.0407 n=522	r=−0.1001 p=0.4239 n=66	r=−0.0491 p=0.2581 n=533	r=−0.0935 p=0.1120 n=290	r=−0.0869 p=0.0486 n=516	r=−0.0379 p=0.4672 n=371	r=−0.1009 p=0.0220 n=515
Stemness (mRNA)	r=0.1709 p=0.0371 n=149	r=0.1939 p=1.0e−05 n=510	r=NA p=NA NA	r=0.0008 p=0.9849 n=507	r=0.1961 p=0.0009 n=283	r=0.1481 p=0.0008 n=511	r=0.0668 p=0.2058 n=361	r=0.1853 p=2.7e−05 n=507
Stemness (DNA methylation)	r=−0.0010 p=0.9944 n=49	r=0.1755 p=6.8e−05 n=510	r=NA p=NA NA	r=−0.1151 p=0.0442 n=306	r=−0.0896 p=0.1434 n=268	r=−0.0869 p=0.0497 n=511	r=−0.0592 p=0.2616 n=361	r=0.1154 p=0.0144 n=449
Predicted pCR to T/FAC chemotherapy	r=0.1877 p=0.0219 n=149	r=−0.0908 p=0.0393 n=516	r=NA p=NA NA	r=−0.0914 p=0.0362 n=525	r=−0.1698 p=0.0038 n=288	r=−0.2020 p=4.0e−06 n=513	r=0.0953 p=0.0695 n=364	r=0.0364 p=0.4116 n=512
Proliferation score	r=0.0435 p=0.5985 n=149	r=0.1981 p=6.6e−06 n=510	r=NA p=NA NA	r=−0.0448 p=0.3142 n=507	r=0.1232 p=0.0404 n=277	r=−0.2725 p=3.8e−10 n=511	r=0.1090 p=0.0401 n=355	r=0.0447 p=0.3422 n=454

	LUSC	MESO	OV	PAAD	PCPG	PRAD	READ	SARC
Autophagy−related prognostic signature	r=0.0050 p=0.9107 n=503	r=0.1291 p=0.2334 n=87	r=−0.1219 p=0.0333 n=305	r=−0.0905 p=0.2285 n=179	r=−0.0133 p=0.8592 n=181	r=−0.0777 p=0.0832 n=498	r=0.0325 p=0.6832 n=160	r=−0.1292 p=0.0374 n=260
Stemness (mRNA)	r=0.1747 p=0.0001 n=484	r=0.1658 p=0.1248 n=87	r=0.1188 p=0.0402 n=299	r=0.1447 p=0.0715 n=156	r=0.1681 p=0.0320 n=163	r=−0.1137 p=0.0119 n=488	r=−0.0498 p=0.5395 n=154	r=0.0251 p=0.6925 n=250
Stemness (DNA methylation)	r=0.2196 p=2.6e−05 n=360	r=0.0723 p=0.5057 n=87	r=−0.3167 p=0.4064 n=9	r=0.0826 p=0.3051 n=156	r=−0.0911 p=0.2474 n=163	r=0.0290 p=0.5228 n=488	r=−0.2023 p=0.0531 n=92	r=0.1307 p=0.0385 n=251
Predicted pCR to T/FAC chemotherapy	r=0.0922 p=0.0395 n=499	r=0.0846 p=0.4362 n=87	r=0.0974 p=0.0913 n=302	r=−0.1188 p=0.1141 n=178	r=−0.1166 p=0.1370 n=164	r=−0.0672 p=0.1364 n=492	r=−0.0807 p=0.3138 n=158	r=0.0746 p=0.2346 n=256
Proliferation score	r=0.1366 p=0.0026 n=484	r=0.1262 p=0.2554 n=83	r=−0.0545 p=0.3751 n=267	r=0.0012 p=0.9883 n=151	r=0.0225 p=0.7755 n=163	r=0.0628 p=0.2096 n=401	r=−0.0470 p=0.5626 n=154	r=−0.0228 p=0.7369 n=220

	SKCM	STAD	TGCT	THCA	THYM	UCEC	UCS	UVM
Autophagy−related prognostic signature	r=−0.0204 p=0.6584 n=472	r=0.0572 p=0.2453 n=415	r=−0.1188 p=0.1477 n=150	r=−0.0092 p=0.8349 n=513	r=−0.5049 p=4.1e−09 n=120	r=−0.0190 p=0.6613 n=532	r=−0.0140 p=0.9177 n=57	r=0.0021 p=0.9852 n=80
Stemness (mRNA)	r=−0.0680 p=0.1411 n=470	r=0.1976 p=7.0e−05 n=399	r=0.4659 p=2.1e−09 n=149	r=0.2167 p=9.5e−07 n=502	r=0.7146 p=7.2e−20 n=119	r=0.1905 p=1.2e−05 n=521	r=0.1036 p=0.4475 n=56	r=−0.1968 p=0.0802 n=80
Stemness (DNA methylation)	r=0.1038 p=0.0244 n=470	r=0.1355 p=0.0092 n=368	r=0.5791 p=1.0e−14 n=149	r=−0.1216 p=0.0064 n=502	r=−0.4554 p=2.0e−07 n=119	r=0.1426 p=0.0038 n=411	r=0.1881 p=0.1651 n=56	r=−0.0573 p=0.6137 n=80
Predicted pCR to T/FAC chemotherapy	r=−0.0778 p=0.0922 n=470	r=0.1706 p=0.0009 n=375	r=0.2667 p=0.0010 n=150	r=−0.0447 p=0.3161 n=504	r=0.5249 p=8.9e−10 n=119	r=−0.0840 p=0.0548 n=524	r=0.2002 p=0.1390 n=56	r=0.1308 p=0.2477 n=80
Proliferation score	r=−0.1607 p=0.0005 n=470	r=0.1990 p=7.8e−05 n=389	r=0.2844 p=0.0004 n=149	r=−0.1713 p=0.0001 n=502	r=0.4992 p=7.5e−09 n=119	r=0.0739 p=0.0919 n=521	r=−0.1412 p=0.2992 n=56	r=−0.3327 p=0.0026 n=80

Spearman_r
1.0
0.5
0.0
−0.5
−1.0
