# Supplementary material for: Targeting BRF2 in Cancer Using Repurposed Drugs
Source: Cancers (Basel). 2021 Jul 27;13(15):3778. doi: 10.3390/cancers13153778 (PMC8345145; doi:10.3390/cancers13153778)
Supplement: Supplementary file 1 [file cancers-13-03778-s001.zip › Analysis/BRF2_1c_Hypoxia_angiogenesis_heatmap3_allstats.pdf]

Hypoxia and angiogenesis (BRF2)

Cancer type

	ACC	BLCA	BRCA	CESC	CHOL	COAD	ESCA	GBM	HNSC	KICH
Angiogenesis score	r=0.0390 p=0.7348 n=78	r=−0.0726 p=0.1488 n=397	r=−0.1279 p=2.4e−05 n=1083	r=0.1053 p=0.0687 n=300	r=0.0202 p=0.9084 n=35	r=0.0091 p=0.8497 n=441	r=−0.1377 p=0.0707 n=173	r=0.0059 p=0.9412 n=156	r=−0.0797 p=0.0712 n=514	r=−0.3836 p=0.0016 n=65
Hypoxia score (Winter)	r=NA p=NA NA	r=0.0143 p=0.7768 n=394	r=0.1096 p=0.0003 n=1068	r=0.0996 p=0.0826 n=305	r=NA p=NA NA	r=0.0986 p=0.0995 n=280	r=NA p=NA NA	r=0.2968 p=0.0002 n=149	r=−0.0630 p=0.1509 n=522	r=NA p=NA NA
Hypoxia score (West)	r=NA p=NA NA	r=−0.0175 p=0.7337 n=382	r=0.0440 p=0.1555 n=1043	r=0.0613 p=0.2917 n=298	r=NA p=NA NA	r=0.0505 p=0.4128 n=265	r=NA p=NA NA	r=0.1400 p=0.0919 n=146	r=−0.1043 p=0.0176 n=517	r=NA p=NA NA
Hypoxia score (Sorensen)	r=NA p=NA NA	r=−0.0159 p=0.7586 n=375	r=−0.1469 p=1.9e−06 n=1041	r=0.0106 p=0.8596 n=279	r=NA p=NA NA	r=0.0501 p=0.4212 n=260	r=NA p=NA NA	r=0.0748 p=0.3677 n=147	r=−0.0234 p=0.6068 n=487	r=NA p=NA NA
Hypoxia score (Seigneuric)	r=NA p=NA NA	r=0.0031 p=0.9504 n=408	r=−0.1112 p=0.0002 n=1100	r=−0.0817 p=0.1541 n=306	r=NA p=NA NA	r=−0.1609 p=0.0064 n=286	r=NA p=NA NA	r=−0.1486 p=0.0649 n=155	r=0.0242 p=0.5807 n=522	r=NA p=NA NA
Hypoxia score (Ragnum)	r=NA p=NA NA	r=−0.0036 p=0.9435 n=398	r=0.0871 p=0.0045 n=1060	r=−0.0211 p=0.7135 n=306	r=NA p=NA NA	r=0.0264 p=0.6598 n=280	r=NA p=NA NA	r=0.0904 p=0.2796 n=145	r=0.0854 p=0.0538 n=511	r=NA p=NA NA
Hypoxia score (Hu)	r=NA p=NA NA	r=−0.0674 p=0.1744 n=408	r=0.0229 p=0.4474 n=1100	r=0.0514 p=0.3700 n=306	r=NA p=NA NA	r=0.0129 p=0.8277 n=286	r=NA p=NA NA	r=0.0086 p=0.9157 n=155	r=−0.0028 p=0.9489 n=522	r=NA p=NA NA
Hypoxia score (Elvidge)	r=NA p=NA NA	r=−0.0762 p=0.1272 n=402	r=−0.1879 p=5.3e−10 n=1076	r=−0.0415 p=0.4736 n=300	r=NA p=NA NA	r=−0.0985 p=0.1000 n=280	r=NA p=NA NA	r=−0.0851 p=0.2925 n=155	r=−0.0974 p=0.0276 n=512	r=NA p=NA NA
Hypoxia score (Buffa)	r=NA p=NA NA	r=0.0257 p=0.6051 n=408	r=0.1513 p=4.6e−07 n=1100	r=0.1363 p=0.0170 n=306	r=NA p=NA NA	r=0.1104 p=0.0621 n=286	r=NA p=NA NA	r=0.3686 p=2.4e−06 n=155	r=−0.0100 p=0.8203 n=522	r=NA p=NA NA

	KIRC	KIRP	LGG	LIHC	LUAD	LUSC	MESO	OV	PAAD	PCPG
Angiogenesis score	r=−0.0289 p=0.5131 n=515	r=0.2182 p=0.0002 n=279	r=−0.0362 p=0.4127 n=514	r=−0.0286 p=0.5881 n=362	r=−0.0829 p=0.0768 n=457	r=−0.1387 p=0.0021 n=487	r=−0.2240 p=0.0418 n=83	r=−0.1449 p=0.0172 n=270	r=0.0735 p=0.3696 n=151	r=0.0167 p=0.8249 n=178
Hypoxia score (Winter)	r=0.1395 p=0.0016 n=510	r=0.2762 p=2.7e−06 n=280	r=0.0925 p=0.0356 n=516	r=0.1286 p=0.0134 n=369	r=0.1503 p=0.0007 n=500	r=0.1055 p=0.0182 n=501	r=NA p=NA NA	r=0.0664 p=0.2586 n=291	r=0.0053 p=0.9447 n=171	r=0.0287 p=0.7009 n=181
Hypoxia score (West)	r=−0.0256 p=0.5630 n=513	r=0.0365 p=0.5545 n=265	r=0.1149 p=0.0093 n=512	r=0.0557 p=0.2923 n=360	r=0.0621 p=0.1734 n=482	r=0.1037 p=0.0211 n=494	r=NA p=NA NA	r=−0.1005 p=0.0956 n=276	r=−0.0977 p=0.2162 n=162	r=0.1142 p=0.1312 n=176
Hypoxia score (Sorensen)	r=−0.0309 p=0.4791 n=526	r=−0.0615 p=0.3274 n=256	r=0.1104 p=0.0145 n=490	r=−0.0321 p=0.5479 n=353	r=0.0500 p=0.2787 n=471	r=0.0990 p=0.0285 n=490	r=NA p=NA NA	r=0.0128 p=0.8322 n=278	r=−0.1195 p=0.1274 n=164	r=−0.0670 p=0.3767 n=176
Hypoxia score (Seigneuric)	r=−0.0074 p=0.8639 n=533	r=−0.1470 p=0.0122 n=290	r=−0.1247 p=0.0046 n=516	r=0.0695 p=0.1816 n=371	r=0.0298 p=0.4995 n=515	r=0.0188 p=0.6748 n=502	r=NA p=NA NA	r=−0.0709 p=0.2176 n=304	r=−0.1632 p=0.0291 n=179	r=−0.2543 p=0.0006 n=181
Hypoxia score (Ragnum)	r=0.0263 p=0.5546 n=508	r=0.0300 p=0.6169 n=281	r=−0.0094 p=0.8329 n=511	r=0.0565 p=0.2914 n=351	r=0.1142 p=0.0106 n=501	r=0.1552 p=0.0005 n=499	r=NA p=NA NA	r=−0.0437 p=0.4601 n=288	r=−0.1558 p=0.0412 n=172	r=−0.0145 p=0.8464 n=181
Hypoxia score (Hu)	r=−0.0220 p=0.6129 n=533	r=0.1099 p=0.0616 n=290	r=0.0272 p=0.5374 n=516	r=0.0006 p=0.9902 n=371	r=0.0238 p=0.5895 n=515	r=0.0088 p=0.8437 n=502	r=NA p=NA NA	r=−0.0515 p=0.3709 n=304	r=−0.0504 p=0.5032 n=179	r=−0.0439 p=0.5571 n=181
Hypoxia score (Elvidge)	r=−0.0151 p=0.7279 n=532	r=−0.0741 p=0.2122 n=285	r=−0.1273 p=0.0039 n=513	r=−0.0542 p=0.2994 n=369	r=−0.1847 p=2.8e−05 n=507	r=−0.0917 p=0.0409 n=497	r=NA p=NA NA	r=−0.2057 p=0.0004 n=295	r=−0.2045 p=0.0062 n=178	r=−0.1834 p=0.0135 n=181
Hypoxia score (Buffa)	r=0.0862 p=0.0466 n=533	r=0.2667 p=4.1e−06 n=290	r=0.1513 p=0.0006 n=516	r=0.1024 p=0.0487 n=371	r=0.1662 p=0.0002 n=515	r=0.2470 p=2.1e−08 n=502	r=NA p=NA NA	r=0.0609 p=0.2895 n=304	r=0.0077 p=0.9190 n=179	r=0.1272 p=0.0879 n=181

	PRAD	READ	SARC	SKCM	STAD	TGCT	THCA	UCEC	UCS	UVM
Angiogenesis score	r=0.0058 p=0.9073 n=405	r=0.0167 p=0.8360 n=156	r=0.0150 p=0.8236 n=223	r=0.1406 p=0.1565 n=103	r=−0.2193 p=1.2e−05 n=391	r=−0.4162 p=1.3e−07 n=149	r=−0.0095 p=0.8330 n=500	r=0.1437 p=0.0009 n=528	r=−0.0892 p=0.5095 n=57	r=−0.0151 p=0.8943 n=80
Hypoxia score (Winter)	r=0.0807 p=0.1424 n=332	r=0.0929 p=0.3919 n=87	r=NA p=NA NA	r=0.0715 p=0.1305 n=449	r=NA p=NA NA	r=NA p=NA NA	r=0.0067 p=0.8804 n=509	r=0.0569 p=0.4586 n=172	r=NA p=NA NA	r=NA p=NA NA
Hypoxia score (West)	r=0.1037 p=0.0595 n=331	r=−0.0068 p=0.9510 n=85	r=NA p=NA NA	r=0.0153 p=0.7542 n=421	r=NA p=NA NA	r=NA p=NA NA	r=−0.0918 p=0.0383 n=509	r=−0.0903 p=0.2575 n=159	r=NA p=NA NA	r=NA p=NA NA
Hypoxia score (Sorensen)	r=−0.0051 p=0.9268 n=328	r=−0.0746 p=0.5001 n=84	r=NA p=NA NA	r=0.0303 p=0.5304 n=432	r=NA p=NA NA	r=NA p=NA NA	r=−0.1475 p=0.0009 n=501	r=−0.0774 p=0.3305 n=160	r=NA p=NA NA	r=NA p=NA NA
Hypoxia score (Seigneuric)	r=0.1712 p=0.0017 n=333	r=−0.1226 p=0.2392 n=94	r=NA p=NA NA	r=−0.0451 p=0.3288 n=471	r=NA p=NA NA	r=NA p=NA NA	r=−0.1684 p=0.0001 n=509	r=0.0015 p=0.9838 n=177	r=NA p=NA NA	r=NA p=NA NA
Hypoxia score (Ragnum)	r=0.0135 p=0.8061 n=331	r=0.0066 p=0.9507 n=91	r=NA p=NA NA	r=−0.0817 p=0.0894 n=433	r=NA p=NA NA	r=NA p=NA NA	r=0.0535 p=0.2281 n=509	r=0.0237 p=0.7574 n=173	r=NA p=NA NA	r=NA p=NA NA
Hypoxia score (Hu)	r=−0.0638 p=0.2455 n=333	r=−0.1249 p=0.2303 n=94	r=NA p=NA NA	r=0.0411 p=0.3730 n=471	r=NA p=NA NA	r=NA p=NA NA	r=0.0270 p=0.5438 n=509	r=−0.0458 p=0.5448 n=177	r=NA p=NA NA	r=NA p=NA NA
Hypoxia score (Elvidge)	r=0.0175 p=0.7530 n=326	r=−0.0653 p=0.5341 n=93	r=NA p=NA NA	r=−0.0279 p=0.5472 n=467	r=NA p=NA NA	r=NA p=NA NA	r=−0.2986 p=9.8e−12 n=499	r=−0.1523 p=0.0442 n=175	r=NA p=NA NA	r=NA p=NA NA
Hypoxia score (Buffa)	r=0.0548 p=0.3184 n=333	r=−0.0203 p=0.8463 n=94	r=NA p=NA NA	r=0.0350 p=0.4481 n=471	r=NA p=NA NA	r=NA p=NA NA	r=0.1321 p=0.0028 n=509	r=0.0730 p=0.3343 n=177	r=NA p=NA NA	r=NA p=NA NA

Spearman_r: 1.0, 0.5, 0.0, −0.5, −1.0
