# Supplementary material for: Targeting BRF2 in Cancer Using Repurposed Drugs
Source: Cancers (Basel). 2021 Jul 27;13(15):3778. doi: 10.3390/cancers13153778 (PMC8345145; doi:10.3390/cancers13153778)

Immune cell infiltration (BRF2)

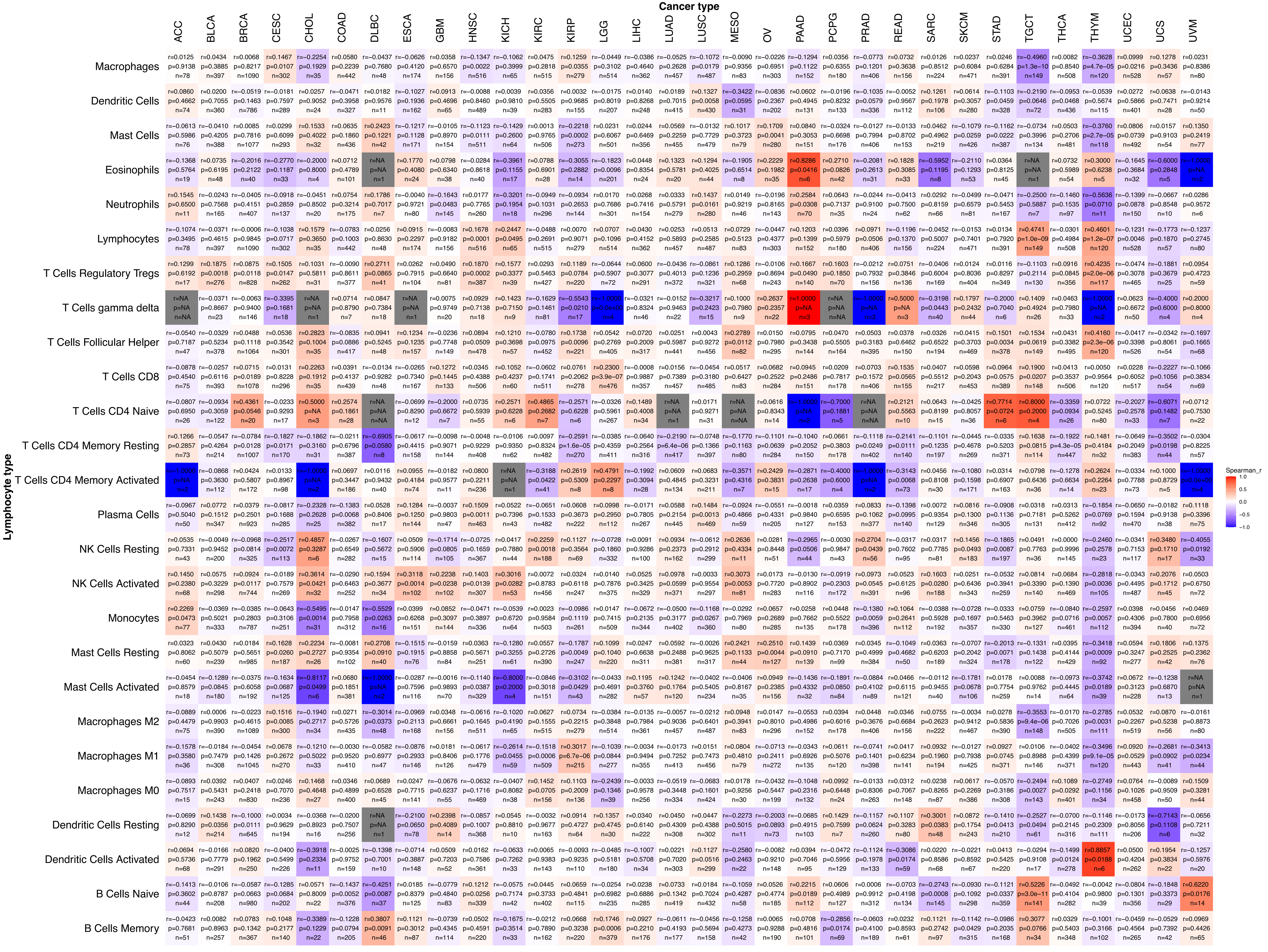

Supplement: Supplementary file 1 [file cancers-13-03778-s001.zip › Analysis/BRF2_1d_Immune_cell_infiltration_heatmap3_allstats.pdf]
