# Supplementary material for: Targeting BRF2 in Cancer Using Repurposed Drugs
Source: Cancers (Basel). 2021 Jul 27;13(15):3778. doi: 10.3390/cancers13153778 (PMC8345145; doi:10.3390/cancers13153778)
Supplement: Supplementary file 1 [file cancers-13-03778-s001.zip › Analysis/BRF2_1e_Neoantigens_cancer_testis_antigens_heatmap3_allstats.pdf]

Neoantigens and cancer testis antigens (BRF2)

Cancer type

	ACC	BLCA	BRCA	CESC	CHOL	COAD	DLBC	ESCA	GBM	HNSC	KIRC
Cancer/testis (CT) antigen load	r=0.1201<br>p=0.3014<br>n=76	r=−0.0171<br>p=0.7357<br>n=391	r=0.0060<br>p=0.8462<br>n=1057	r=0.0553<br>p=0.3596<br>n=277	r=−0.1896<br>p=0.2754<br>n=35	r=0.0355<br>p=0.4700<br>n=416	r=NA<br>p=NA<br>n=NA	r=0.0473<br>p=0.5367<br>n=173	r=0.1338<br>p=0.1051<br>n=148	r=0.1064<br>p=0.0163<br>n=509	r=0.0426<br>p=0.3398<br>n=505
Indel neoantigen load	r=−0.1723<br>p=0.2753<br>n=42	r=0.0952<br>p=0.0738<br>n=354	r=0.0531<br>p=0.1569<br>n=713	r=0.0989<br>p=0.1802<br>n=185	r=−0.0481<br>p=0.8193<br>n=25	r=0.1066<br>p=0.0599<br>n=312	r=−0.0661<br>p=0.7238<br>n=31	r=NA<br>p=NA<br>n=NA	r=0.1183<br>p=0.2898<br>n=82	r=0.0501<br>p=0.3220<br>n=392	r=−0.0336<br>p=0.5512<br>n=317
SNV neoantigen load	r=0.0560<br>p=0.6307<br>n=76	r=−0.0377<br>p=0.4519<br>n=401	r=0.0423<br>p=0.1855<br>n=982	r=0.0041<br>p=0.9455<br>n=275	r=0.0535<br>p=0.7603<br>n=35	r=0.0636<br>p=0.2261<br>n=364	r=0.0905<br>p=0.5942<br>n=37	r=0.0908<br>p=0.2862<br>n=140	r=−0.1133<br>p=0.1810<br>n=141	r=0.0943<br>p=0.0395<br>n=477	r=−0.1183<br>p=0.0278<br>n=346

	KIRP	LGG	LIHC	LUAD	LUSC	MESO	OV	PAAD	PCPG	PRAD
Cancer/testis (CT) antigen load	r=−0.0983<br>p=0.1043<br>n=274	r=0.1206<br>p=0.0066<br>n=506	r=0.0525<br>p=0.3278<br>n=349	r=0.0771<br>p=0.1058<br>n=441	r=0.1379<br>p=0.0025<br>n=477	r=0.1536<br>p=0.1709<br>n=81	r=0.0435<br>p=0.4800<br>n=266	r=0.0553<br>p=0.5017<br>n=150	r=0.1568<br>p=0.0456<br>n=163	r=0.1231<br>p=0.0136<br>n=401
Indel neoantigen load	r=0.0704<br>p=0.2705<br>n=247	r=0.0783<br>p=0.1629<br>n=319	r=−0.1004<br>p=0.0809<br>n=303	r=−0.0131<br>p=0.7827<br>n=443	r=0.0698<br>p=0.1446<br>n=439	r=0.0328<br>p=0.8122<br>n=55	r=NA<br>p=NA<br>n=NA	r=0.1570<br>p=0.1489<br>n=86	r=0.0117<br>p=0.9503<br>n=31	r=0.0479<br>p=0.4404<br>n=262
SNV neoantigen load	r=−0.0487<br>p=0.4291<br>n=266	r=0.0370<br>p=0.4082<br>n=502	r=0.0462<br>p=0.3933<br>n=343	r=0.0877<br>p=0.0512<br>n=495	r=0.0597<br>p=0.2020<br>n=458	r=0.2404<br>p=0.0340<br>n=78	r=−0.1045<br>p=0.1929<br>n=157	r=−0.0293<br>p=0.7160<br>n=157	r=−0.0476<br>p=0.5541<br>n=157	r=0.0546<br>p=0.2311<br>n=482

	READ	SARC	SKCM	STAD	TGCT	THCA	THYM	UCEC	UCS	UVM
Cancer/testis (CT) antigen load	r=0.0417<br>p=0.6164<br>n=147	r=0.1131<br>p=0.0942<br>n=220	r=−0.0912<br>p=0.3571<br>n=104	r=0.0353<br>p=0.5013<br>n=365	r=0.2250<br>p=0.0092<br>n=133	r=−0.0890<br>p=0.0464<br>n=501	r=NA<br>p=NA<br>n=NA	r=0.0180<br>p=0.6823<br>n=519	r=0.0063<br>p=0.9635<br>n=55	r=−0.1952<br>p=0.0827<br>n=80
Indel neoantigen load	r=0.0656<br>p=0.5148<br>n=101	r=−0.0757<br>p=0.3900<br>n=131	r=0.0887<br>p=0.4720<br>n=68	r=NA<br>p=NA<br>n=NA	r=0.0840<br>p=0.5127<br>n=63	r=−0.1037<br>p=0.2723<br>n=114	r=0.2238<br>p=0.1144<br>n=51	r=0.0580<br>p=0.2554<br>n=387	r=0.1645<br>p=0.2980<br>n=42	r=0.1961<br>p=0.3080<br>n=29
SNV neoantigen load	r=−0.0056<br>p=0.9510<br>n=121	r=0.0612<br>p=0.3565<br>n=229	r=0.1929<br>p=0.0498<br>n=104	r=0.2685<br>p=0.0085<br>n=95	r=−0.0567<br>p=0.5030<br>n=142	r=0.0304<br>p=0.5120<br>n=469	r=−0.2940<br>p=0.0014<br>n=115	r=0.0677<br>p=0.1296<br>n=503	r=−0.3449<br>p=0.0099<br>n=55	r=0.1517<br>p=0.1938<br>n=75

Spearman_r
1.0
0.5
0.0
−0.5
−1.0
