# Supplementary material for: Targeting BRF2 in Cancer Using Repurposed Drugs
Source: Cancers (Basel). 2021 Jul 27;13(15):3778. doi: 10.3390/cancers13153778 (PMC8345145; doi:10.3390/cancers13153778)
Supplement: Supplementary file 1 [file cancers-13-03778-s001.zip › Analysis/BRF2_1g_Signal_transduction_pathway_scores_heatmap3_allstats.pdf]

Signal Transduction Pathways (BRF2)

Cancer type (columns) × Signal Transduction Pathway (rows); colour scale: Spearman_r (−1.0 to 1.0)

Signal Transduction Pathway	BLCA	BRCA	COAD	GBM	HNSC	KIRC	LUAD	LUSC	OV	READ	UCEC
Hormone	r=−0.1719 p=0.0542 n=126	r=0.0377 p=0.3019 n=753	r=−0.0644 p=0.2452 n=327	r=0.0960 p=0.4256 n=71	r=0.0441 p=0.5235 n=212	r=0.0144 p=0.7605 n=451	r=0.0564 p=0.3906 n=234	r=−0.0510 p=0.4816 n=193	r=0.0427 p=0.5224 n=227	r=0.0155 p=0.8654 n=122	r=−0.0773 p=0.1267 n=392
TSC mTOR	r=0.0294 p=0.7435 n=126	r=0.1728 p=1.9e−06 n=753	r=−0.1315 p=0.0173 n=327	r=−0.2344 p=0.0491 n=71	r=−0.0033 p=0.9617 n=212	r=−0.1485 p=0.0016 n=451	r=−0.0318 p=0.6285 n=234	r=0.3014 p=2.1e−05 n=193	r=0.0530 p=0.4265 n=227	r=−0.1677 p=0.0648 n=122	r=0.0283 p=0.5771 n=392
RTK score	r=−0.1767 p=0.0477 n=126	r=−0.1328 p=0.0003 n=753	r=−0.0685 p=0.2167 n=327	r=0.0916 p=0.4472 n=71	r=−0.1787 p=0.0091 n=212	r=−0.0468 p=0.3214 n=451	r=−0.0852 p=0.1941 n=234	r=−0.0701 p=0.3328 n=193	r=0.0387 p=0.5617 n=227	r=−0.0140 p=0.8783 n=122	r=−0.0847 p=0.0939 n=392
Ras MAPK	r=−0.2836 p=0.0013 n=126	r=−0.1166 p=0.0013 n=753	r=−0.0777 p=0.1611 n=327	r=−0.0459 p=0.7040 n=71	r=−0.0704 p=0.3077 n=212	r=−0.0949 p=0.0440 n=451	r=−0.0712 p=0.2780 n=234	r=0.0257 p=0.7229 n=193	r=0.0310 p=0.6427 n=227	r=−0.1972 p=0.0295 n=122	r=−0.1448 p=0.0041 n=392
PI3K Akt	r=−0.0926 p=0.3025 n=126	r=−0.0751 p=0.0395 n=753	r=−0.0397 p=0.4744 n=327	r=−0.2901 p=0.0141 n=71	r=0.0923 p=0.1808 n=212	r=−0.0337 p=0.4757 n=451	r=0.0568 p=0.3870 n=234	r=0.0446 p=0.5381 n=193	r=0.0666 p=0.3181 n=227	r=−0.1595 p=0.0793 n=122	r=0.0411 p=0.4165 n=392
Hormone b	r=0.1177 p=0.1894 n=126	r=0.0151 p=0.6782 n=753	r=−0.0449 p=0.4188 n=327	r=−0.0776 p=0.5203 n=71	r=0.0310 p=0.6535 n=212	r=0.0549 p=0.2447 n=451	r=−0.0584 p=0.3736 n=234	r=0.0543 p=0.4531 n=193	r=−0.0354 p=0.5954 n=227	r=0.2708 p=0.0026 n=122	r=−0.1206 p=0.0169 n=392
EMT score	r=−0.1010 p=0.2605 n=126	r=−0.0669 p=0.0664 n=753	r=0.0250 p=0.6518 n=327	r=−0.0743 p=0.5382 n=71	r=−0.0102 p=0.8829 n=212	r=−0.0372 p=0.4311 n=451	r=−0.0286 p=0.6635 n=234	r=0.0824 p=0.2545 n=193	r=−0.0953 p=0.1523 n=227	r=0.0652 p=0.4755 n=122	r=0.0811 p=0.1088 n=392
DNA damage response	r=−0.0340 p=0.7054 n=126	r=−0.0055 p=0.8802 n=753	r=0.0395 p=0.4766 n=327	r=0.0327 p=0.7869 n=71	r=0.0114 p=0.8691 n=212	r=0.0709 p=0.1328 n=451	r=0.0632 p=0.3357 n=234	r=−0.0120 p=0.8687 n=193	r=0.0092 p=0.8907 n=227	r=0.0743 p=0.4159 n=122	r=−0.0569 p=0.2611 n=392
Combined reactive	r=−0.1652 p=0.0646 n=126	r=−0.0320 p=0.3808 n=753	r=−0.0281 p=0.6124 n=327	r=−0.0318 p=0.7923 n=71	r=−0.0649 p=0.3467 n=212	r=−0.0557 p=0.2380 n=451	r=0.0136 p=0.8360 n=234	r=0.1178 p=0.1029 n=193	r=−0.1203 p=0.0705 n=227	r=0.0110 p=0.9042 n=122	r=0.0623 p=0.2182 n=392
Cell cycle	r=−0.0913 p=0.3093 n=126	r=0.1133 p=0.0018 n=753	r=0.0791 p=0.1535 n=327	r=0.0140 p=0.9077 n=71	r=0.0516 p=0.4546 n=212	r=−0.0044 p=0.9260 n=451	r=0.1436 p=0.0280 n=234	r=−0.0111 p=0.8783 n=193	r=−0.0728 p=0.2748 n=227	r=−0.0473 p=0.6051 n=122	r=0.1207 p=0.0168 n=392
Breast reactive	r=−0.2123 p=0.0170 n=126	r=−0.0033 p=0.9272 n=753	r=−0.0908 p=0.1010 n=327	r=−0.0023 p=0.9846 n=71	r=−0.1007 p=0.1438 n=212	r=−0.0331 p=0.4827 n=451	r=0.0117 p=0.8593 n=234	r=0.0970 p=0.1795 n=193	r=−0.1189 p=0.0738 n=227	r=−0.0661 p=0.4693 n=122	r=0.0440 p=0.3848 n=392
Apoptosis	r=0.0220 p=0.8066 n=126	r=−0.0589 p=0.1066 n=753	r=0.0727 p=0.1895 n=327	r=0.0906 p=0.4525 n=71	r=−0.0079 p=0.9090 n=212	r=−0.0468 p=0.3209 n=451	r=0.1032 p=0.1154 n=234	r=0.0170 p=0.8149 n=193	r=−0.0917 p=0.1686 n=227	r=0.1430 p=0.1162 n=122	r=0.0464 p=0.3597 n=392

Spearman_r: 1.0, 0.5, 0.0, −0.5, −1.0
