# Supplementary material for: Targeting BRF2 in Cancer Using Repurposed Drugs
Source: Cancers (Basel). 2021 Jul 27;13(15):3778. doi: 10.3390/cancers13153778 (PMC8345145; doi:10.3390/cancers13153778)
Supplement: Supplementary file 1 [file cancers-13-03778-s001.zip › cancers-1262527_Supplementary_proofread_R1.pdf]

Supplementary Materials:

Cancer type abbreviations: ACC, Adrenocortical Cancer; BLCA, Bladder Cancer; BRCA, Breast Cancer; CESC, Cervical Cancer; CHOL, Cholangiocarcinoma (bile duct cancer); COAD, Colon Cancer; DLBC, Large B-cell Lymphoma; ESCA, Esophageal Cancer; GBM, Glioblastoma; HNSC, Head and Neck Cancer; KICH, Kidney Chromophobe; KIRC, Kidney Clear Cell Carcinoma; KIRP, Kidney Papillary Cell Carcinoma; LGG, Lower Grade Glioma; LIHC, Liver Cancer; LUAD, Lung Adenocarcinoma; LUSC, Lung Squamous Cell Carcinoma; MESO, Mesothelioma; OV, Ovarian Cancer; PAAD, Pancreatic Cancer; PCPG, Pheochromocytoma & Paraganglioma; PRAD, Prostate Cancer; READ, Rectal Cancer; SARC, Sarcoma; SKCM, Melanoma; STAD, Stomach Cancer; TGCT, Testicular Cancer; THCA, Thyroid Cancer; THYM, Thymoma; UCEC, Endometrioid Cancer; UCS, Uterine Carcinosarcoma; UVM, Ocular melanoma.

Supplementary Tables:

Table S1: Binding energies of top-ranking docked compounds from Drug Bank against C361 amino acid of BRF2.

**Drugs**	**Binding-energy (kCal/mol)**
Cetrorelix	-10.3
Goserelin	-9.8
Nafarelin	-9.7
Degarelix	-9.6
Ganirelix	-9.6
Fertagyl	-9.4
Nilotinib	-9.3
Lumacaftor	-8.9
Desmopressin	-8.8

Table S2: Binding energies of top-ranking docked compounds from NCI against C361 amino acid of BRF2.

**Drugs**	**Binding-energy (kCal/mol)**
Nilotinib	-9.3
Imatinib	-8.2
Sonidegib	-8
Dabrafenib	-7.9
Osimertinib	-7.8
Vismodegib	-7.7
Niraparib	-7.6
Rucaparib	-7.6
Crizotinib	-7.3

Ribociclib	-7.2
Celecoxib	-7.1
Methotrexate	-6.6

Supplementary figure legends

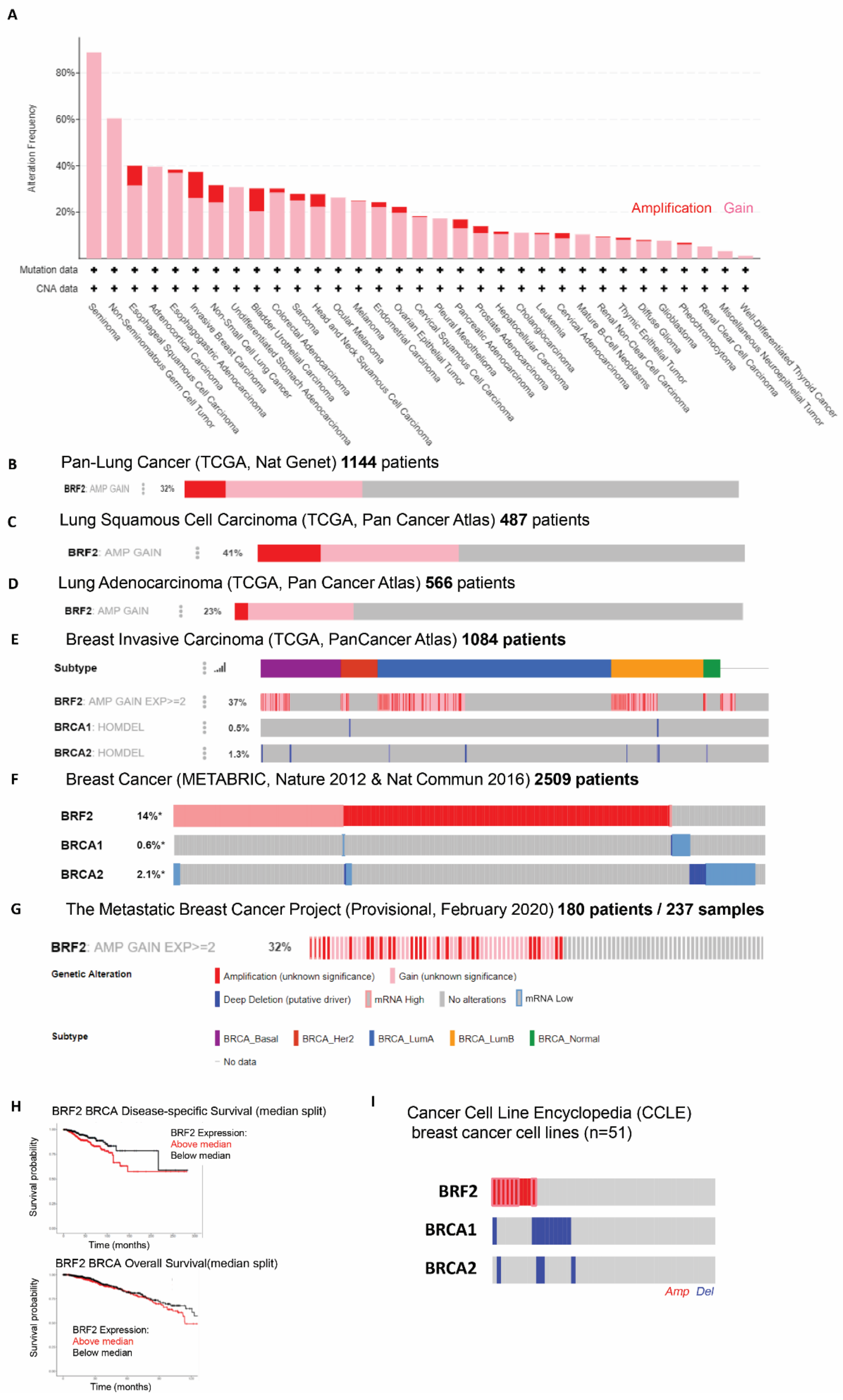
A
Alteration Frequency
80%
60%
40%
20%
Amplification
Gain
Mutation data
CNA data
Seminoma
Non-Seminomatous Germ Cell Tumor
Esophageal Squamous Cell Carcinoma
Adrenocortical Carcinoma
Esophagogastric Adenocarcinoma
Invasive Breast Carcinoma
Non-Small Cell Lung Cancer
Undifferentiated Stomach Adenocarcinoma
Bladder Urothelial Carcinoma
Colorectal Adenocarcinoma
Sarcoma
Head and Neck Squamous Cell Carcinoma
Ocular Melanoma
Melanoma
Endometrial Carcinoma
Ovarian Epithelial Tumor
Cervical Squamous Cell Carcinoma
Pleural Mesothelioma
Pancreatic Adenocarcinoma
Prostate Adenocarcinoma
Hepatocellular Carcinoma
Cholangiocarcinoma
Leukemia
Cervical Adenocarcinoma
Mature B-Cell Neoplasms
Renal Non-Clear Cell Carcinoma
Thymic Epithelial Tumor
Diffuse Glioma
Glioblastoma
Pheochromocytoma
Renal Clear Cell Carcinoma
Miscellaneous Neuroepithelial Tumor
Well-Differentiated Thyroid Cancer
B
Pan-Lung Cancer (TCGA, Nat Genet) 1144 patients
BRF2: AMP GAIN 32%
C
Lung Squamous Cell Carcinoma (TCGA, Pan Cancer Atlas) 487 patients
BRF2: AMP GAIN 41%
D
Lung Adenocarcinoma (TCGA, Pan Cancer Atlas) 566 patients
BRF2: AMP GAIN 23%
E
Breast Invasive Carcinoma (TCGA, PanCancer Atlas) 1084 patients
Subtype
BRF2: AMP GAIN EXP>=2 37%
BRCA1: HOMDEL 0.5%
BRCA2: HOMDEL 1.3%
F
Breast Cancer (METABRIC, Nature 2012 & Nat Commun 2016) 2509 patients
BRF2 14%*
BRCA1 0.6%*
BRCA2 2.1%*
G
The Metastatic Breast Cancer Project (Provisional, February 2020) 180 patients / 237 samples
BRF2: AMP GAIN EXP>=2 32%
Genetic Alteration
Amplification (unknown significance)
Gain (unknown significance)
Deep Deletion (putative driver)
mRNA High
No alterations
mRNA Low
Subtype
BRCA_Basal
BRCA_Her2
BRCA_LumA
BRCA_LumB
BRCA_Normal
No data
H
BRF2 BRCA Disease-specific Survival (median split)
Survival probability
BRF2 Expression:
Above median
Below median
Time (months)
BRF2 BRCA Overall Survival(median split)
Survival probability
BRF2 Expression:
Above median
Below median
Time (months)
I
Cancer Cell Line Encyclopedia (CCLE)
breast cancer cell lines (n=51)
BRF2
BRCA1
BRCA2
Amp
Del

Figure S1. (A): Chart shows percent of alteration frequency (copy number alteration) for amplification (red) or copy number gain (pink) of *BRF2* across various cancers using the cBioPortal for Cancer Genomics. **(B-G)**: Oncoprint of cBioPortal analysis shows amplification (red) or gain of function (pink) in BRF2 for indicated cancer, dataset, and number of patients. Oncoprints from the TCGA and METABRIC breast cancer datasets showing mutually exclusive alterations between BRF2 and BRCA1/2, with upregulation of BRF2 rarely observed in tumors with BRCA1/2 loss or downregulation. (**H**): Kaplan-Meier survival analysis for TCGA breast cancer patients for Disease-specific survival (upper) and Overall survival (lower). Patients were split into two groups, with high and low BRF2 expression levels, split using the median BRF2 expression level as the cut-off. Numbers of patients at risk are shown at the bottom. (**I**): The amplification or gain of function in BRF2 comparing with BRCA1 and/or BRCA2 loss in breast cancer cell lines from Cancer Cell Line Encyclopedia (CCLE) (n=51, Mann-Whitney U test).

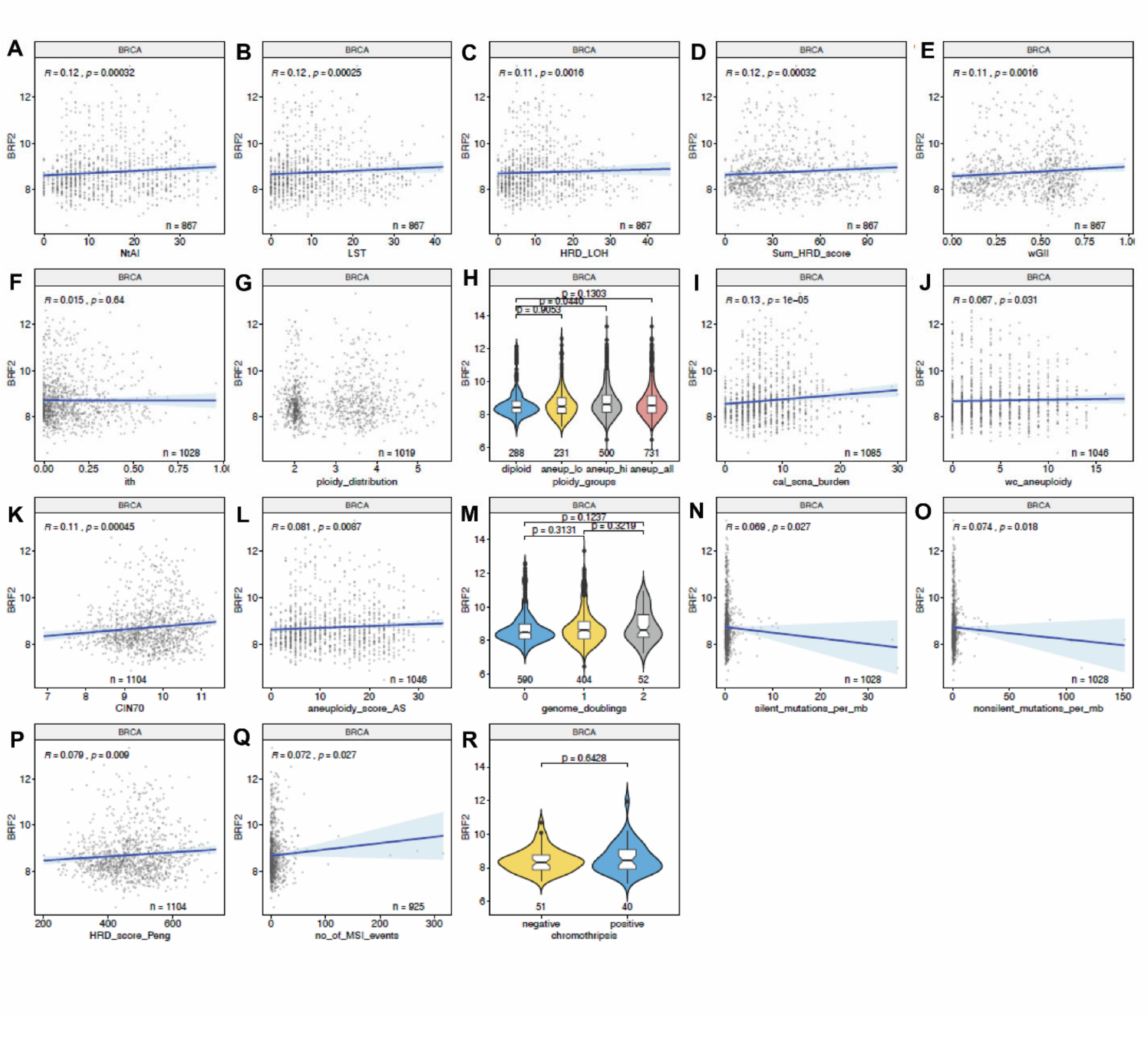

Figure S2. A-R: The analyses show correlations between gene expression (from The Cancer genome Atlas (TCGA) RNAseq datasets) and 18 measures of genomic instability (GI). The 18 measures are labelled "a" through "r". For all panels, the y-axis shows the relative expression level

of BRF2. Details on each of the GI variables on the x-axis of each panel is available in methods. In all scatter plots, linear regression analysis was performed and R and p values are given according to Spearman's rank correlation. Samples sizes, n, are also included. Panel (g) intentionally does not include linear regression analysis, because there is no scientific value for that for this data type. Instead, these data were binned and included in panel (h). Violin plots in panels (h), (m) and (r) show p values calculated according to Mann-Whitney U tests.

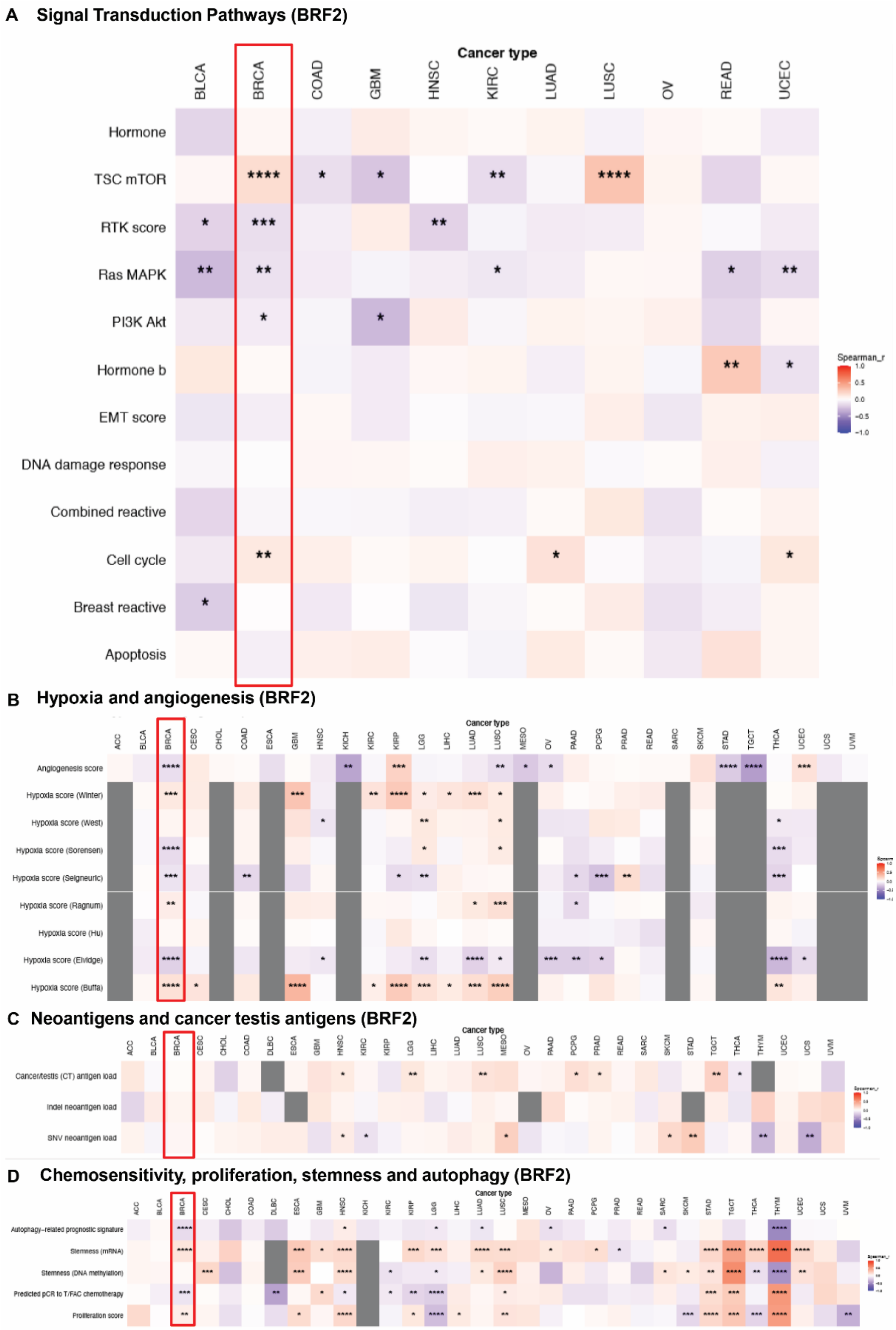

Figure S3. A-D: The heatmaps show the correlations between gene expression levels and the variables listed on the y-axis in up to 32 cancer types, whose abbreviations are listed on the top x-axis. For more details see the methods. Each tile in the heatmaps shows the Spearman correlation

between the gene expression levels (mRNA levels from The Cancer Genome Atlas (TCGA) RNAseq datasets)) and the variables listed below. The color of each tile reflects the Spearman correlation coefficient (r), as indicated by the color key on the right. Each tile shows the Spearman p value abbreviations in the following format: blank tiles: $p > 0.05$; *, $p < 0.05$; **, $p < 0.01$; ***, $p < 0.001$; ****, $p < 0.0001$.

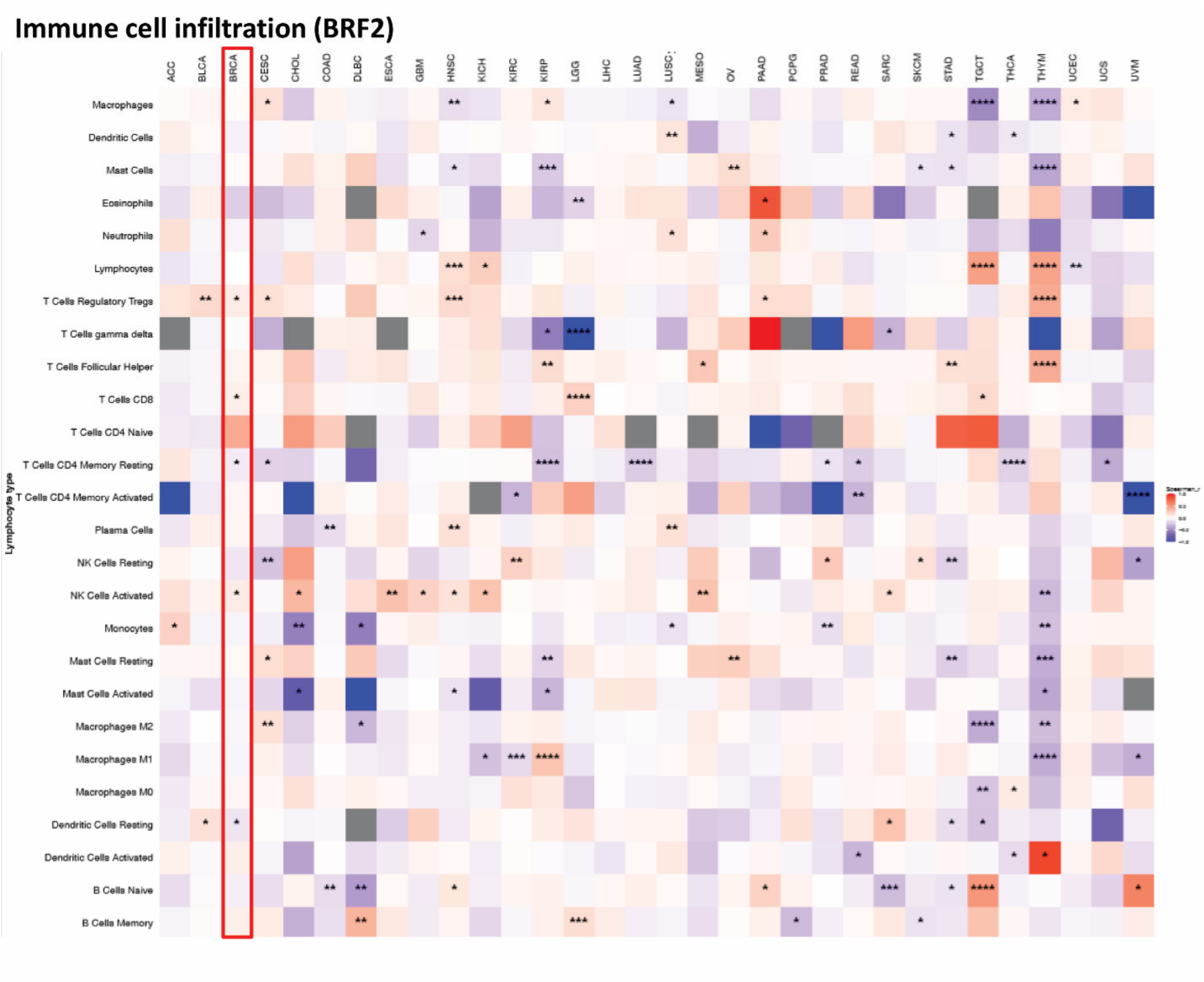

Figure S4. Immune cell infiltration: The levels of indicated tumour-infiltrated immune cell types were estimated using the tumor immune estimation resource, for more details see the methods and for statistics methodology see Sup 3.

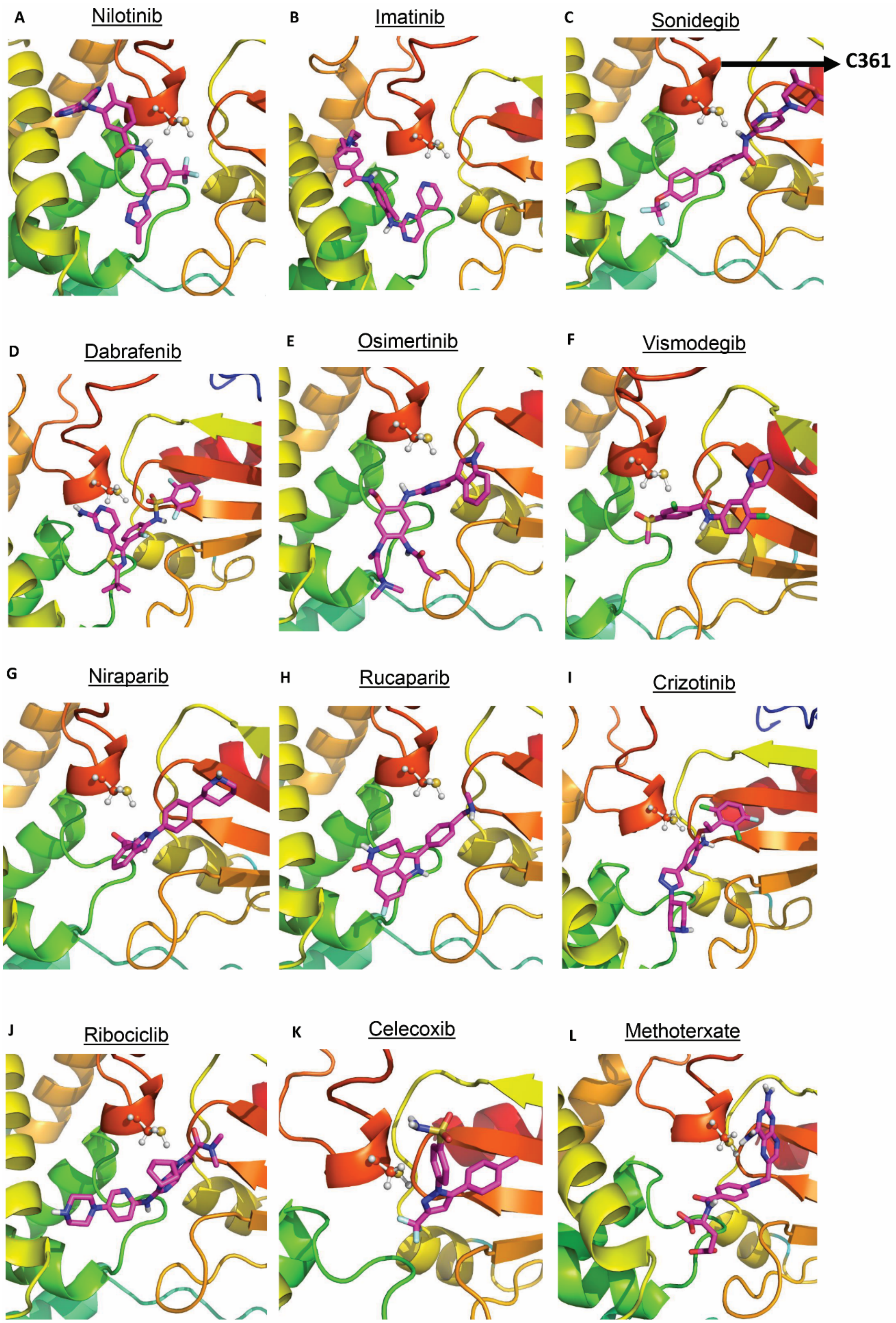
A
Nilotinib
B
Imatinib
C
Sonidegib
C361
D
Dabrafenib
E
Osimertinib
F
Vismodegib
G
Niraparib
H
Rucaparib
I
Crizotinib
J
Ribociclib
K
Celecoxib
L
Methoterxate

Figure S5. A-L: Docking shows architecture of binding of indicated drugs to C361 amino acid of BRF2 (black arrowed).

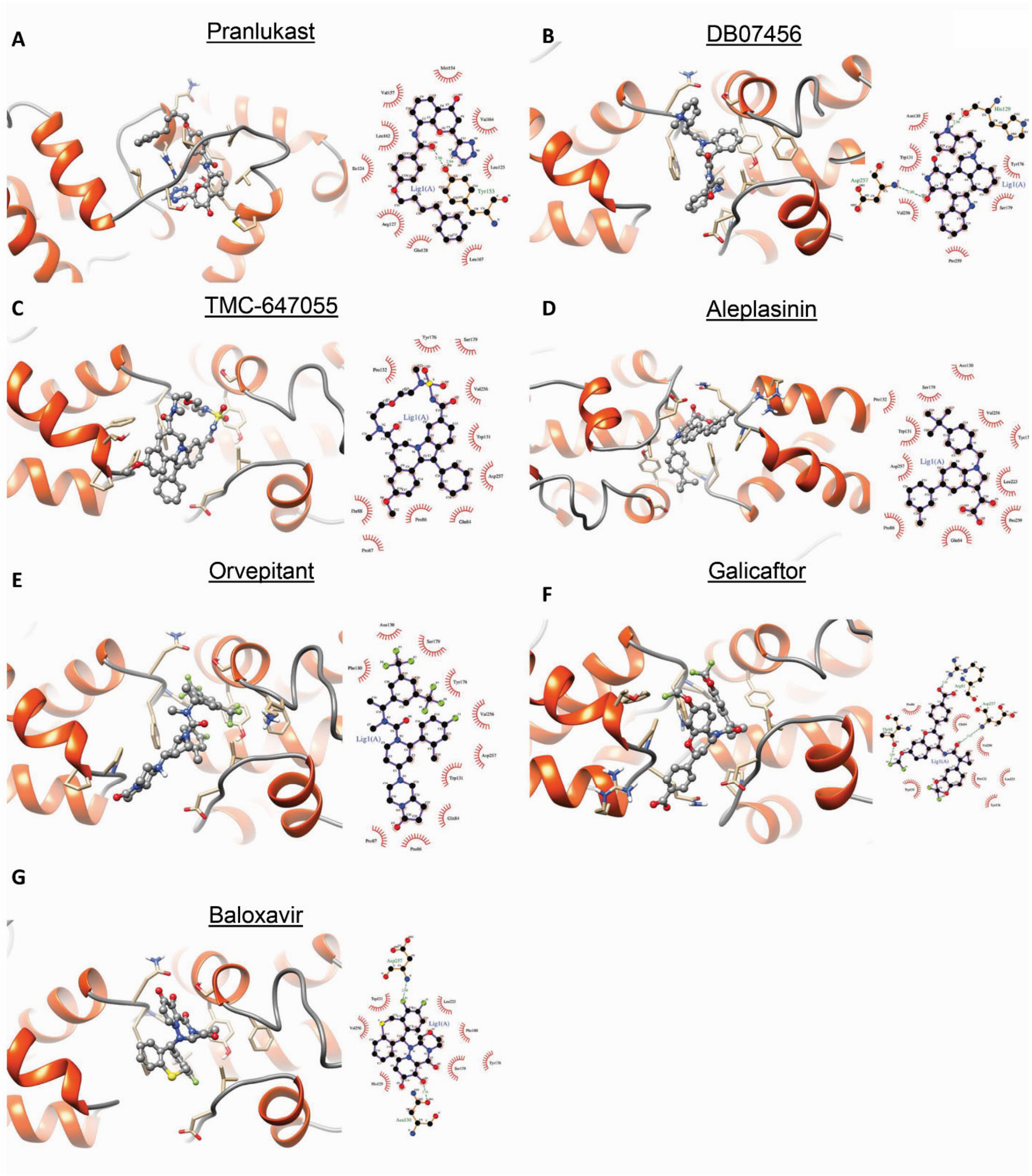

Figure S6. A-E. Molecular docking and interaction between amino acids of BRF2 with ligands as indicated in figure (compounds listed 8th -15th in ranking).

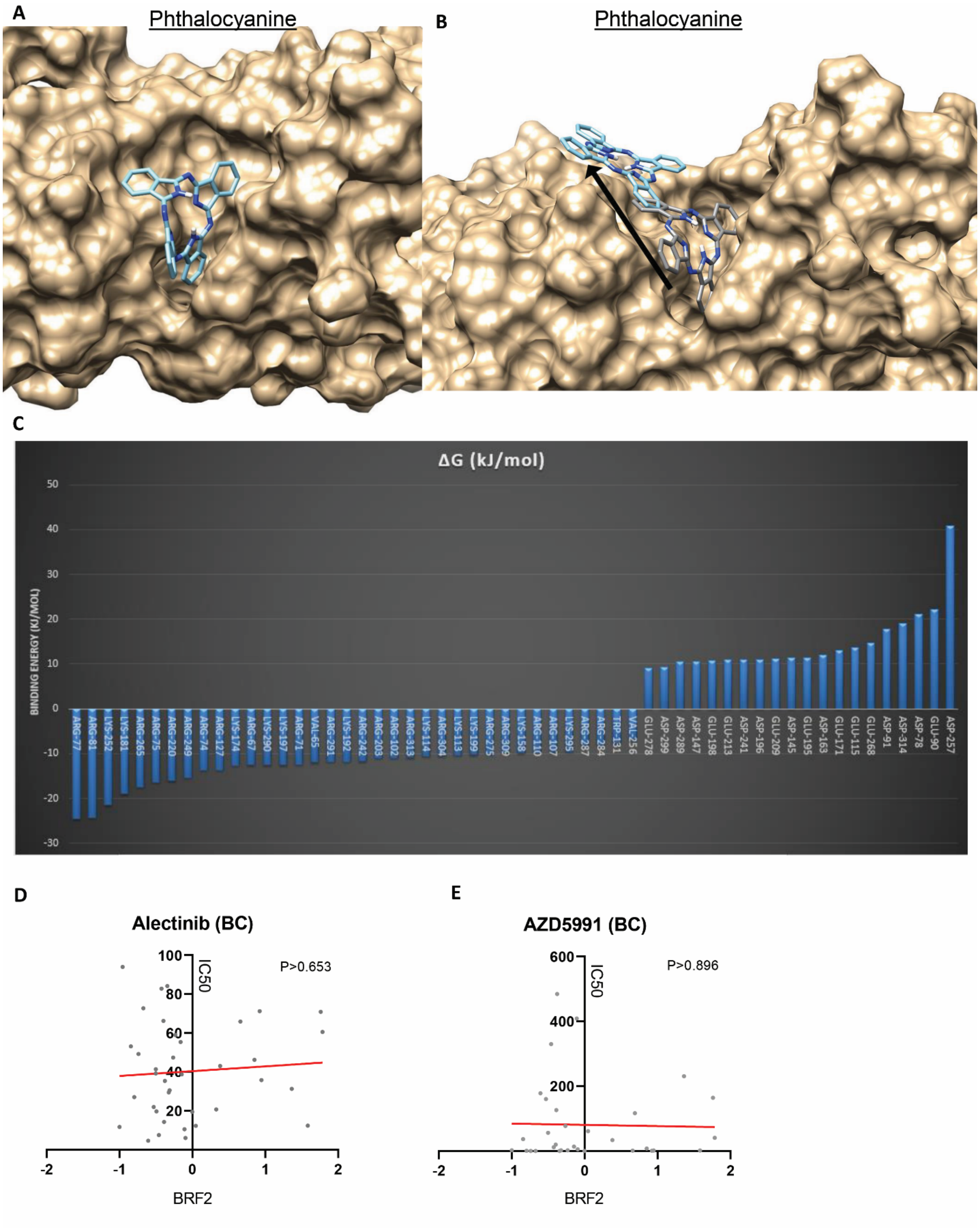

Figure S7. A-D. The surface view of docked BRF2-Phthalocyanine complex at **(A)** starting point **(B)** 8 ns after simulation. The arrow shows the direction of ligand in moving out of the cavity. **(C)** The Electrostatic energy calculated for all residues. **(D-E)** Correlation between BRF2 expression

in breast cancer cell lines (threshold of expression is set be-tween -2 to 2) and IC50 of **(D)** Alectinib and **(E)** AZD5991.
